# New Strategies for the Treatment of Older Myeloma Patients

**DOI:** 10.3390/cancers15102693

**Published:** 2023-05-10

**Authors:** Alessandra Larocca, Lorenzo Cani, Giuseppe Bertuglia, Benedetto Bruno, Sara Bringhen

**Affiliations:** 1 Division of Hematology, Department of Molecular Biotechnology and Health Sciences, University of Torino, 10126 Torino, Italy; 2 SSD Clinical Trial in Oncoematologia e Mieloma Multiplo, Department of Oncology, Azienda Ospedaliero-Universitaria Città della Salute e della Scienza di Torino, 10126 Torino, Italy; 3 Division of Hematology, Azienda Ospedaliero-Universitaria Città della Salute e della Scienza di Torino, 10126 Torino, Italy

**Keywords:** multiple myeloma, elderly patients, frailty

## Abstract

**Simple Summary:**

While novel therapies have improved outcomes in multiple myeloma (MM), physicians are calling for greater caution when managing this hematologic malignancy in older patients due to their fragility, which increases their vulnerability to toxic events. Additionally, this patient population may be excluded from clinical trials due to comorbidities, whereby available data are not always applicable in real-word clinical practice. This review delves into available frailty assessment tools that can be used to identify patients who are unfit or frail and tailor therapy to achieve better outcomes while minimizing toxicity. Current therapeutic strategies for managing transplant-ineligible patients with newly diagnosed MM and relapsed or refractory MM are also described, with the aim of guiding physicians when selecting treatment options.

**Abstract:**

Multiple myeloma (MM) mostly affects older patients, who represent a highly heterogeneous population. In the last few years, the introduction of novel agents led to a significant improvement in the outcome of MM patients. Nonetheless, this positive trend is less likely to occur in all older patients due to comorbidities/disabilities and major susceptibility to toxic events. Furthermore, older patients with major comorbidities are usually excluded or underrepresented in most registrational clinical trials. In this context, physicians have called for greater caution in the management of the disease. Several scores allow for the identification of frail and unfit patients and establish the possibility of tailoring therapy, reducing toxicity. This review explores the available tools for the assessment of frailty and what has been done to improve the discriminative power of the available scores. Thereafter, it describes the main therapeutic strategies for the management of transplant-ineligible (NTE) newly diagnosed (ND) MM patients and relapsed/refractory (RR) MM patients, in order to better guide physicians in choosing treatment options and to suggest possible strategies for more frail patients.

## 1. Introduction

Multiple myeloma (MM) is a hematologic malignancy that typically occurs in older patients. The median age at the time of diagnosis is 69 years, and more than 30% of patients are older than 75 years [1].

Although new therapies have improved median survival, reaching approximately 6 years [2], the available data suggest that age is a major parameter affecting outcome, which worsens decade by decade, reaching about 28.9 months of median overall survival (OS) in patients aged 80 years and older [3]. The impact of age on survival is also highlighted in many major clinical trials, although the selection of patients enrolled in these studies does not totally reflect the characteristics of the real-world population [3,4,5].

In addition to chronological age, other factors such as the incidence and severity of comorbidities, functional impairments, and independence status are strong predictors of life expectancy [6], which is extremely variable in the same age group, thus suggesting that not only chronological age is important, but also health status.

Although the trend in MM research has always been to increase the efficacy of the therapy by combining different drugs (doublets, triplets, and quadruplets), this does not always translate into a benefit, particularly in more frail patients, who are more sensitive to possible adverse toxicities [7].

Furthermore, the efficacy of therapies tends to decrease during the course of the disease, while the proportion of patients receiving therapies at each subsequent line also constantly decreases. In a study published by Yong and colleagues, including 22% of patients over 75 years old, 62% of patients received second-line treatment, and <25% actually received three lines of therapy [8]. This concept was also highlighted by Fonseca e al. in their study, which showed that transplant-ineligible (NTE) patients who received just one line of therapy were significantly older and exhibited a higher incidence of comorbidities [9].

This premise underlines the necessity of choosing the optimal treatment for each older patient according to their characteristics.

## 2. Frailty: Definition and Tools

In recent years, the concept of “frailty” has become an important clinical and decision-making factor in managing older MM patients. The cumulative decline in physiological systems depletes homeostatic reserves and results in frailty, which is a state of increased vulnerability and reduced ability to restore homeostasis after a stressful event [10].

Frailty increases with age, ranging from about 20% in patients aged 75–79 years to 50% in those aged >85 years [11]. MM and its related symptoms (anemia, renal disease, bone disease, and immune dysfunction) may accelerate age-related physiological decompensation and contribute to the aging process [12,13].

For older cancer patients, the International Society of Geriatric Oncology (SIOG) recommends a Comprehensive Geriatric Assessment (CGA), which is a multidimensional, interdisciplinary diagnostic tool for the assessment of comorbidities, social status, functional status, mental state, polypharmacy, nutritional status, and the presence of geriatric syndromes [14]. Unlike the more subjective performance status (PS), CGA is an objective measure of patient conditions and provides substantial information about the functional status of older oncologic and hematologic patients [15], as well as of those with a good PS. Indeed, as reported by Repetto et al. [16], the assessment of functional status with the widely used Karnofsky PS or Eastern Cooperative Oncology Group (ECOG) PS is less effective in older patients than in the adult population due to the interference of comorbidities. Furthermore, GA may complement and add substantial information to the physician’s clinical judgment. There are no specific data available for MM patients, while a small study of 173 lymphoma patients, which aimed to demonstrate the value of GA, effectively showed the superiority of geriatric evaluation over clinical judgment alone, particularly in non-fit patients [15].

Unfortunately, a full comprehensive GA is often considered a time-consuming procedure that is difficult to use in everyday clinical practice [17]. Thus, over the last few years, different simplified frailty scores have been proposed and developed in MM patients to better define their frailty and fitness status.

The current gold-standard frailty score was introduced by the International Myeloma Working Group (IMWG) and was based on age, functional status assessed by the Activities of Daily Living (ADL) and Instrumental ADL (IADL) scales, and Charlson Comorbidity Index (CCI). The ADL and IADL scores assess basic and more advanced self-care activities, tasks of household management, and independence status, while the CCI is used to estimate the number and severity of comorbidities. According to the IMWG frailty score, patients were classified as fit (0 points), intermediate-fit (1 point), or frail (≥2 points). These three categories were associated with different rates of progression-free survival (PFS), OS, and risk of toxicity. In particular, frail patients showed inferior OS and PFS rates and a higher incidence of nonhematologic adverse events (AEs) and drug discontinuation. Furthermore, in a multivariate analysis, the IMWG frailty score was found to be independent of intrinsic characteristics of the disease, such as International Staging System (ISS) stage, chromosomal abnormalities, and type of treatment [18].

There are still many issues to be resolved before having a more reliable tool to define frailty. One of them is the role of age. Even though age does not necessarily define biological frailty, patients aged >80 years are determined to be frail according to the IMWG score. Indeed, a subanalysis of frail patients enrolled in the EMN01, 26866138-MMY2069, and IST-CAR-506 studies recently showed that survival was similar among patients determined to be frail by age only and those determined to be frail for any other reason (such as the impairments defined by ADL and IADL or the presence of comorbidities) [19].

A redefinition of frailty was recently proposed by Stege and colleagues, including 71 intermediate-fit and 130 frail patients with symptomatic newly diagnosed (ND)MM enrolled in the HOVON-123 study and treated with a dose-adjusted melphalan–prednisone–bortezomib (MPV) regimen. Two revised frailty indices (RFI and RFI-2) were derived from the original IMWG frailty score by defining intermediate-fit patients (total frailty score = 1–2) and frail patients (total frailty score ≥ 3). For the RFI-2, 1 point was also added in the case of CCI  ≥ 3, giving more importance to comorbidities. Despite the limited number of patients, this revised cutoff for frailty led to the identification of a smaller yet more vulnerable frail population with inferior outcomes [20].

An additional frailty score named Revised Myeloma Comorbidity Index (R-MCI) was developed by Engelhardt et al. Unlike the IMWG frailty score, which included only patients who were NTE, the R-MCI was developed in a younger population (median age of 63 years) and included patients who were eligible for autologous stem-cell transplantation (ASCT). In the multivariate analysis, five risk factors (renal and pulmonary impairment, PS, frailty, and age) were found to be significant in terms of OS. The R-MCI was defined by combining these factors and adding cytogenetic characteristics, thereby allowing the identification of fit (score ≤ 3), intermediate-fit (score = 4–6), and frail patients (score > 6) [21].

In the FIRST study [22], Facon et al. proposed a simplified version of the IMWG frailty score by identifying two categories with different outcomes: non-frail and frail. In addition to retrospective CCI and age, this simplified score also incorporated the ECOG PS, which is assessed by physicians [23]. This score was then validated by another study (HOVON87/NMSG18) with 612 NTE patients. Although there was no difference in terms of PFS between non-frail and frail patients, probably explained by the fact that non-frail patients more often received second-line treatment, frail patients had a higher risk of death and nonhematologic AEs of grade 3–4 [24].

The Mayo Clinic risk score, which included levels of N-terminal fragment of the type B natriuretic peptide (NT-proBNP) [25], and the Myeloma Risk Profile (MRP) score, which incorporated both tumor (ISS stage) and host characteristics (PS, age, and C-reactive protein) [26], have been proposed as valuable clinical tools. To the best of our knowledge, the first score has not yet been validated, whereas the MRP score was retrospectively validated in real-life patients from the Danish Registry [27] and will be prospectively validated and compared to the IMWG Frailty Score in the ongoing UK-MRA Myeloma XIV study (FiTNEss) [28].

An objective biological indicator of frailty that is gaining interest in the geriatric evaluation of patients is sarcopenia, which describes a condition of progressive and generalized decline in muscle mass and function associated with an increased likelihood of adverse outcomes [29]. The prevalence of sarcopenia among people aged ≥65 years is 5–10%, reaching 38% in cancer patients [30]. Additionally, markers of cellular senescence (including markers of DNA damage, telomere length, and cell-cycle arrest) and inflammation associated with aging are being explored. For instance, the capacity of DNA repair decreases with frailty, and parameters of increased DNA breakage (such as increased phosphorylated H2AX levels) can be integrated as objective tools for frailty detection [31].

Given the number of frailty scores, the goal should be to standardize frail assessments in clinical trials to best translate the most effective and sensitive score into clinical practice. In the future, a clinical score will be completed by biological markers.

**Figure 1:**
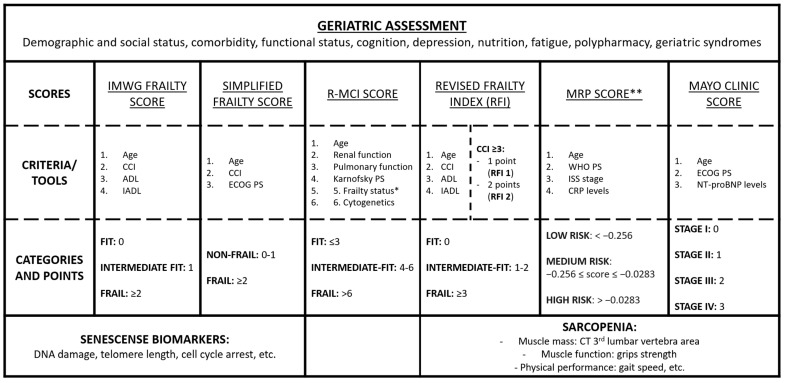
Main tools for geriatric assessment in patients with multiple myeloma. * Frailty status in the R-MCI score includes the Karnofsky index, time up/go, IADL and subjective fitness. ** MRP formula: (PS score − 2) × 0.199 + (age − 74.4) × 0.0165 + (ISS stage − 2) × 0.212 + [log (CRP + 1) − 2.08] × 0.0315. Abbreviations: IMWG, International Myeloma Working Group; CCI, Charlson Comorbidity Index; ADL, Katz Index of Independence in Activities of Daily Living; IADL, Lawton Instrumental Activities of Daily Living; PS, performance status; ECOG, Eastern Cooperative Oncology Group; R-MCI, Revised Myeloma Comorbidity Index; MRP, Myeloma Risk Profile; RFI, Revised Frailty Index; WHO, World Health Organization; ISS, International Staging System; NT-proBNP, circulating N-terminal pro B type natriuretic peptide; CT, computed tomography; CRP, C-reactive protein.

Tools for geriatric assessments are summarized in Figure 1.

## 3. First-Line Treatment for Transplant-Ineligible Patients with Multiple Myeloma

**Figure 2:**
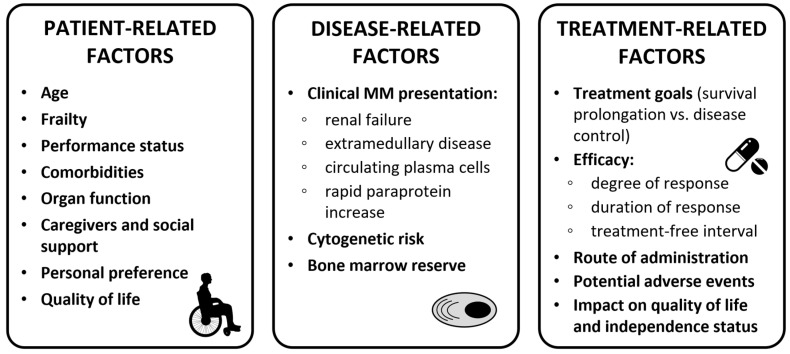
Clinical considerations for treatment decision in transplant-ineligible patients. Abbreviations: MM, multiple myeloma.

When selecting the best first-line treatment for older NDMM patients, several factors need to be considered: patient-related factors (e.g., age, frailty status, organ function, comorbidities, patient preference, and social status), disease-related factors (e.g., renal failure, presence of extramedullary disease [EMD], and presence of high-risk cytogenetics), and treatment-related factors (e.g., efficacy, treatment goals, potential AEs, and impact on quality of life; see Figure 2).

Defining treatment goals for each older patient is crucial. For fit patients, the aim is to achieve good and long-lasting responses, such as complete response (CR) or minimal residual disease (MRD) negativity, using combinations such as triplets or quadruplets. Unlike older fit patients, the main goal in the treatment of intermediate-fit patients is to achieve a balance between efficacy and toxicity, whereas reducing therapy-related toxicities and preserving the best levels of quality of life and independence as long as possible are the key aims for the treatment of frail patients [32,33].

The VISTA trial showed that the bortezomib–melphalan–prednisone (VMP) triplet was superior to the MP doublet in terms of median (m)PFS (24 months vs. 16 months, hazard ratio [HR] 0.48) and median (m)OS (56 months vs. 43 months, HR 0.69), even in patients older than 75 years [34]. Peripheral neuropathy is the major limiting toxicity related to bortezomib, which is mostly administered once a week in clinical practice [35].

The FIRST study compared continuous Rd vs. limited-duration Rd (Rd18; 18 months) vs. melphalan–prednisone–thalidomide (MPT), demonstrating a significant advantage of continuous Rd in terms of mPFS (26 months in the Rd vs. 21 in the Rd18 and MPT arms) [36]. An improvement of 10 months in OS in the continuous Rd arm vs. MPT was also reported, without significant differences vs. Rd18 [22].

Over the last few years, the introduction of the anti-CD38 monoclonal antibody (mAb) daratumumab has substantially changed the treatment landscape for NTE NDMM patients. According to the updated ESMO guidelines, the current standards of care are daratumumab–lenalidomide–dexamethasone (D–Rd), daratumumab–VMP (D–VMP), and bortezomib–lenalidomide–dexamethasone (VRd). If these options are not available, VMP or Rd combinations are recommended [37,38].

Both the MAIA (D–Rd vs. Rd) and the ALCYONE (D–VMP vs. VMP) studies led to the approval of daratumumab as the backbone of first-line therapy. D–Rd and D–VMP were approved by the European Medicines Agency (EMA) in October 2019 on the basis of the results of these studies. D-Rd was associated with a high overall response rate (ORR) of 93% with a 32% of MRD negativity rate [39,40]. In the subanalysis of the MAIA study, D–Rd, as compared with Rd, was shown to increase PFS, OS, and MRD negativity rates in several subgroups, including patients aged ≥75 years, those with renal failure, and those with ISS stage III [41]. In patients aged ≥75 years, the occurrence of grade 3–4 AEs was similar between the D–Rd and Rd arms (95.5% vs. 95%). The most common AEs were neutropenia (D–Rd 62.4% vs. Rd 41.5%), lymphopenia (D–Rd 21.0% vs. Rd 12.6%), and pneumonia (D–Rd 20.4% vs. Rd 14.5%). Despite this, the discontinuation rate due to treatment-emergent (TE)AEs was lower in the D–Rd than in the Rd arm (7.1% vs. 15.9%). Quality of life (QoL) data also showed a benefit from D–Rd over Rd, particularly in terms of physical functioning, fatigue, and median time of pain relief [42].

The ALCYONE study also yielded higher response and MRD negativity rates in the experimental arm vs. the control arm (ORR 91% vs. 74%; MRD negativity 28% vs. 7%). The most common grade ≥ 3 AEs with D–VMP vs. VMP were neutropenia (40% vs. 39%), thrombocytopenia (34% vs. 38%), and infections (23% vs. 15%; with an incidence of pneumonia of 11% vs. 4%) [43].

In a retrospective analysis of the MAIA study on frailty, patients were divided into three categories (fit, intermediate, and frail) using a frailty score [44] that incorporated age, CCI, and ECOG PS evaluation. Fit and intermediate patients had a longer PFS than frail patients, but the PFS benefit from D–Rd vs. Rd was maintained across all the frailty subgroups. The clinical benefit from D–Rd and D–VMP in NTE NDMM patients was not associated with safety issues. Data are shown in Table 1.

However, choosing the most appropriate regimen between D–VMP and D–Rd can be challenging due to the lack of comparison data. Furthermore, the studies that led to the registration of these two combinations included patients with a median age of 73 and 71 years who met specific inclusion criteria. The ongoing Real MM study aims to overcome these limitations. In the first version of the study, real-life, unselected NTE NDMM patients were enrolled and randomized 1:1 to either VMP or Rd. Following the widespread adoption of daratumumab as the backbone of first-line therapy in older patients, the Real MM study was amended to randomize patients to D–VMP vs. D–Rd. Preliminary data on the first 250 patients treated with VMP vs. Rd showed no differences in terms of mPFS (29.6 vs. 26.3 months, HR 0.82, *p* = 0.41), while VMP showed a prolonged mPFS in high-risk patients, as compared with Rd (not reached [NR] vs. 13.3 months, HR 0.22). There was no difference in terms of PFS according to frailty status (calculated using the IMWG frailty score). Adjusted dose upfront and during treatment did not affect efficacy, particularly in the subgroup of frail patients [47]. Additional analysis is needed to determine whether these results are confirmed in terms of OS and whether, as expected, the addition of daratumumab will have an impact on efficacy in all patients and specific subgroups, such as high-risk and frail patients. Unlike many trials investigating the current standards of care, the Real MM trial includes an unselected population, making its results valuable in identifying patients or subgroups that would benefit from this type of therapy (see Table 2) [43,45,48].

In a subanalysis of the Real MM trial on health-related (HR)QoL, VMP treatment was associated with a worse HRQoL compared with Rd. VMP showed a transitory decrease in HRQoL, especially during the early treatment cycles, with an improvement from approximately the sixth month of therapy. In particular, during the twice-weekly bortezomib schedule, VMP was associated with temporary worsening in physical functioning, nausea, appetite, and fatigue. Depth of response did not seem to predict HRQoL in this cohort of real-life NTE MM patients, while frail patients showed relatively lower and long-lasting HRQoL compared with fit patients. The data collected after adding daratumumab will be useful in assessing the impact of treatment on QoL in different subgroups of patients treated with standard-of-care regimens [50].

In April 2019, the EMA approved VRd in NTE NDMM patients older than 65 years based on the results of the SWOG S0777 trial, which showed significantly better PFS (43 vs. 30 months, HR 0.71) and OS (75 vs. 64 months, HR 0.70) with the VRd triplet vs. Rd. In the multivariate analysis, the impact of both the treatment groups was retained regardless of age and intent to transplant. These differences were statistically significant for patients aged <65 years and >75 years. However, in the VRd group, the median OS (mOS) was 65 months in patients aged >65 years vs. NR in patients aged <65 years. Similarly, in the Rd group, median OS was 56 months in patients >65 years vs. 98 months in those aged <65 years. Furthermore, as expected, the incidence of grade 3–4 toxicities was higher in the VRd arm (82% vs. 75%), particularly in the case of grade 3–4 peripheral neuropathy (11% vs. 3%) [5]. In the extended 7-year follow-up, the advantage of VRd vs. Rd in terms of PFS (41 vs. 29 months for Rd) and OS (NR vs. 69 months) was confirmed [48].

A modified version of VRd (VRd-lite) was investigated in a phase II study in NTE patients. The lenalidomide dose was reduced to 15 mg/day, and bortezomib was administered weekly. The median age of the patient population was 73 years, the ORR was 86% with a mPFS of 41.9 months, and treatment was better tolerated, with 2% of grade 3–4 neuropathy. These results suggest that VRd-lite may be a suitable and feasible option for older MM patients [51,52].

In the absence of a head-to-head comparison of different treatment sequences in the new treatment landscape for MM, a study by Fonseca et al. explored different clinical scenarios to determine the clinical value of using daratumumab in NTE NDMM patients in the first-line setting or of saving it for subsequent lines of therapy. The authors compared two different therapy sequencing strategies: (1) D–Rd upfront followed by a pomalidomide- or carfilzomib-based approach at relapse; (2) VRD/Rd upfront followed by a daratumumab-based combination at relapse. D–Rd in first-line treatment improved mOS by 2 years compared with delaying daratumumab-based regimens until the second line after VRd failure (mOS 8.9 vs. 6.9 years) and by 3.2 years after Rd failure (mOS 8.9 vs. 5.75 years). Moreover, a higher probability of being alive at 5, 10, and 15 years was associated with first-line D–Rd, as compared with first-line VRd or Rd. These data suggest the importance of first-line therapy and confirm that achieving the longest possible PFS in first-line therapy drives survival outcomes [53].

Due to the large heterogeneity of older MM patients, a personalized treatment approach according to patient frailty status may be considered. In particular, frail patients may benefit from a gentler approach. The EMN01 study stratified patients according to the IMWG frailty score and showed no differences in terms of PFS and OS in NTE intermediate-fit and frail patients who received Rd vs. melphalan–prednisone–lenalidomide (MPR) vs. cyclophosphamide–prednisone–lenalidomide (CPR). In contrast, fit patients had better outcomes with the MPR triplet (HR 0.72 for PFS of MPR vs. Rd). Thus, this study showed that fit patients could benefit from a triplet, whereas frail patients could benefit from less intensive treatments [54].

The RV-MM-PI-0752 study evaluated a steroid-sparing approach in intermediate-fit patients, comparing continuous Rd treatment (as administered in patients aged >75 years in the FIRST trial [22]) to 9 Rd induction cycles followed by lenalidomide maintenance at a lower dose (10 mg; Rd–R). Notably, in this setting of patients, no differences in PFS or OS were observed between the two treatment strategies, whereas event-free survival (EFS; including a combination of toxicity and efficacy) was significantly longer in the Rd–R arm. Furthermore, Rd–R resulted in better tolerability compared with Rd, particularly in terms of nonhematologic toxicity (grade ≥ 3, 33% vs. 43%) and lenalidomide dose reduction (45% vs. 62%) [55]. These results, together with results of other studies, may help to adapt standard treatment to frail patients, since de-escalation and early dexamethasone interruption do not have a negative impact on outcome.

Another steroid-sparing regimen was explored in NDMM patients who were determined to be frail according to age, comorbidities, and ECOG PS ≥ 2. This study compared Rd vs. daratumumab plus lenalidomide (DR), with only the first 2 cycles of dexamethasone. A preliminary analysis showed that ORRs were 77% vs. 89%, and that the 12-month rates of very good partial response (VGPR) or better were 42% vs. 58% in the Rd vs. DR arms, respectively. As compared with Rd, DR was associated with better rates of MRD negativity at 10^−5^ (3% vs. 10%). The rates of grade ≥ 3 infections and discontinuation were similar in both arms (16% in Rd vs. 13% in DR, not statistically significant). Awaiting longer follow-up and PFS data, this dexamethasone-sparing regimen with daratumumab has shown encouraging responses and good tolerability in this subset of frail patients [56].

Often, study results do not reflect the real-world population due to their strict inclusion and exclusion criteria. The ongoing noninterventional study MM-034 evaluated the efficacy and tolerability of lenalidomide (Len)-based (including Rd and VRd) and non-Len-based (including VMP) regimens in 890 NTE NDMM patients. Patients treated with lenalidomide showed a safety profile similar to those in pivotal trials. More patients discontinued therapy in non-Len-based cohorts than those in Len-based cohorts, and patients who had ≥1 dose adjustment had a longer median duration of response than those who did not. The Len-based cohort showed a nearly double mOS, as compared with the non-Len-based cohort, but patients who discontinued lenalidomide due to AEs experienced a significant worsening of mOS. These data showed that tolerability to treatment was critical for improving patient outcomes [57].

Several studies investigated therapeutic approaches guided by frailty evaluation. The phase II HOVON 143 trial investigated the combination of daratumumab, ixazomib, and low-dose dexamethasone (Ixa–Dara–dex) in NTE NDMM patients who were intermediate-fit and frail according to the IMWG frailty score. The ORR after induction was 71% and 78% in intermediate-fit and frail patients, with a mPFS of 17.4 and 13.8 months and a 12-month OS of 92% and 78%, respectively. Therapy discontinuation occurred in 51% of frail patients, 9% of whom discontinued due to toxicity and 9% of whom discontinued due to death (8% within 2 months, most of whom due to toxicity), while treatment discontinuation due to toxicity was 17% in intermediate-fit patients (6% interrupted the whole regimen, while 11% interrupted ixazomib only). Ixa–Dara–dex is, therefore, a feasible combination in older patients, although treatment discontinuation due to toxicity and early mortality negatively affected PFS and OS, consequently remaining a concern, especially in frail patients [58,59].

The ongoing FiTNEss (UK-MRA Myeloma XIV) trial is investigating a concept of frailty-adjusted dosing by incorporating a first-line treatment including ixazomib–lenalidomide–dexamethasone (Ixa–Rd). In the experimental arm, therapy is adjusted based on the IMWG frailty score, as compared with a non-frailty-adjusted induction in the control arm. The study is ongoing, and results are awaited to understand which approach is most effective and appropriate [28].

Ongoing studies are evaluating newer and more complex first-line combinations in NTE older patients (see Table 3).

## 4. Treatment of Relapsed/Refractory Patients with Multiple Myeloma

The availability of new compounds and their combinations in multidrug regimens has provided MM patients with new options. However, it has also presented new challenges for physicians, particularly for the treatment of frail patients. Currently, few data support the choice of the best treatment strategy for intermediate-fit and frail patients at relapse. Data guiding the current treatment approach for relapsed/refractory (RR)MM patients mostly derive from subgroup analyses in clinical trials, which often report the outcomes of patients on the basis of age (<75 vs. ≥75 years).

Treatment decisions at relapse should be driven by patient and disease factors and, above all, by previous therapies and potential refractoriness to a drug or a drug class. Currently, the early use of an anti-CD38 mAb in the frontline setting (especially in the D-Rd schedule, with daratumumab until disease progression) is reducing its availability in the subsequent lines of therapy, as patients are already refractory to it.

A Rd-based regimen is recommended for patients who received a bortezomib-based therapy upfront without lenalidomide (e.g., VMP and D–VMP). Four clinical trials showed that the addition of daratumumab, carfilzomib, ixazomib, and elotuzumab to the Rd doublet led to improved outcomes.

In the POLLUX study (D–Rd vs. Rd), daratumumab confirmed improved outcomes, even in patients over 75 years of age (mPFS: 28.9 with D-Rd vs. 11.4 months with Rd, HR 0.27). Regarding safety, grade 3–4 TEAEs occurred in 86.2% vs. 91.9% of patients aged 65–74 years, respectively. Neutropenia was the most common grade 3–4 TEAE in patients aged 65–74 years, as well as in those aged ≥75 years [65,66].

In the ASPIRE trial, carfilzomib–lenalidomide–dexamethasone (KRd) was associated with significantly longer PFS in patients aged ≥75 years, as compared with Rd (mPFS 30.3 vs. 16.6 months, HR 0.62). However, older patients receiving KRd experienced a higher rate of cardiovascular toxicity, as compared with younger patients (grade 3 AEs 14% vs. 5%, respectively) [67,68].

In the TOURMALINE-MM1 trial, ixazomib in combination with Rd (Ixa–Rd) also showed an improvement in median PFS vs. Rd in patients older than 75 years, although not statistically significant (18.5 vs. 13.1 months, HR 0.87) [69].

In the ELOQUENT-2 trial, the addition of elotuzumab to Rd (Elo-Rd) resulted in a PFS and OS advantage over Rd that was also observed in all clinically relevant subgroups, including 20% of patients aged >75 years (mOS: 48.5 vs. 27.4 months, HR 0.69), making this triplet a valid option for older patients who were not exposed to lenalidomide in the first line of therapy [70,71,72].

Of note, all these studies enrolled patients with previous exposure to lenalidomide, showing similar results to those reported in lenalidomide-naïve patients, but they did not enroll lenalidomide-refractory patients. A class shift to regimens containing a proteasome inhibitor (PI) is, therefore, recommended in lenalidomide-refractory patients.

In patients primarily exposed and refractory to lenalidomide-based regimens such as D–Rd or Rd, combinations based on bortezomib or carfilzomib are the backbone of salvage therapies.

The CASTOR study showed improved outcomes in patients treated with daratumumab–bortezomib–dexamethasone (D–Vd) vs. Vd, even in patients over 75 years of age (mPFS: 17.9 with D–Vd vs. 8.1 months with Vd, HR 0.26). Regarding the safety profile, the incidence of grade 3–4 TEAEs in patients aged ≥75 years was 90% vs. 81.9% in patients aged 65–74 years. Thrombocytopenia was the most common grade 3–4 TEAE in both the age groups [66].

The ENDEAVOR study showed the benefits of carfilzomib–dexamethasone (Kd) vs. Vd in terms of PFS (19 vs. 9 months, HR 0.38) and OS (36.1 vs. 23.9 months, HR 0.78) in older patients over 75 years of age [73,74], who, however, also experienced a higher rate of grade 3 carfilzomib-related most frequent AEs (anemia, thrombocytopenia, hypertension, and pneumonia) compared with younger patients (10.4% vs. 3.6%) [73,74]. These data suggest that carfilzomib could be a viable treatment option for lenalidomide-refractory older patients, after a careful analysis (and possibly a correction) of cardiovascular risk.

Notably, in both the ENDEAVOR and the CASTOR trials, the mPFS observed with Kd and D–Vd in patients refractory to lenalidomide (9 and 8 months, respectively) was inferior to that observed in the overall population (19 and 17 months, respectively).

In the phase III IKEMA trial, which included 32% of patients refractory to lenalidomide, the addition of isatuximab to carfilzomib and dexamethasone (Isa–Kd) vs. Kd significantly improved PFS and MRD negativity rates (29.6% vs. 13.0%), while maintaining a manageable safety profile regardless of age. Indeed, a subgroup analysis of the IKEMA study analyzed efficacy and safety in patients aged <70 and ≥70 years, showing improved PFS rates with isatuximab in both groups (HR 0.609 and 0.386, respectively), while the rate of grade ≥ 3 AEs was slightly higher in patients aged ≥70 years. The most common AEs were hypertension and pneumonia [75,76].

A step forward in the treatment of lenalidomide-refractory patients is represented by pomalidomide-based regimens. In the phase III OPTIMISMM study, enrolling 70% of patients with lenalidomide-refractory disease, the mPFS in lenalidomide-refractory patients receiving pomalidomide–bortezomib–dexamethasone (PVd) was 18 months [77,78]. In a subanalysis conducted according to the frailty score proposed by Facon et al. [23], frail patients experienced more grade 3 AEs and treatment discontinuations with PVd vs. Vd. Notwithstanding this, frail RRMM patients administered PVd demonstrated superior PFS and ORR rates compared with those administered Vd, consistently with the outcomes observed in the overall population [79].

In the phase III ICARIA-MM trial, isatuximab was combined with pomalidomide and dexamethasone (Isa–Pd) vs. Pd, showing a significantly longer mPFS with Isa–Pd in all subgroups, including patients aged ≥75 years (11.4 vs. 4.5 months, HR 0.49). Patients aged ≥75 years experienced higher rates of grade 3–4 AEs, while the rate of treatment discontinuation was similar to that observed in younger patients. By contrast, infusion-related reactions (IRRs) were less frequent in patients aged ≥75 years than in younger patients.

In the ELOQUENT-3 trial, the addition of elotuzumab to Pd (Elo–Pd) was associated with a PFS advantage over Pd in patients refractory to lenalidomide, including 22% of patients over 75 years of age (HR 0.62) [80].

Daratumumab was investigated in combination with pomalidomide and dexamethasone (D–Pd) in RRMM patients enrolled in the APOLLO trial. At a median follow-up of 16.9 months, a superior PFS was observed in the D–Pd group, as compared with the Pd group (mPFS 12.4 vs. 6.9 months, HR 0.63). At the last update, after a median follow-up of 39.6 months, the mOS was longer in the D–Pd arm than in the Pd arm (34.4 vs. 23.7 months, HR 0.82). Of note, 42% of patients were 65–75 years old and 8% >75 years old [81,82]. Furthermore, the subcutaneous (sc) formulation of daratumumab was associated with a reduced rate of IRRs and a substantially shorter duration of administration, as compared with the intravenous (IV) formulation [82].

The treatment of patients who are triple-class refractory to immunomodulatory drugs, PIs, and anti-CD38 mAbs remains a challenge, with a mOS of 9 months and few available therapeutic alternatives [83,84]. B-cell maturation antigen (BCMA)-targeting agents represent highly effective salvage therapies for triple-class refractory patients and include innovative mechanisms of actions: antibody–drug conjugates (ADCs), bispecific antibodies (bsAbs), and chimeric antigen receptor (CAR) T-cell therapy. However, the feasibility, safety, and optimization of these therapies (particularly of bsAbs and CAR T-cell therapy) in older patients are currently under investigation.

Recently, the noninterventional, retrospective ALFA study focused on the efficacy and safety of belantamab mafodotin, confirming the results of the DREAMM-2 trial in a real-world population. The median age was 70 years, with 30% of patients aged ≥75 years. Fifty-eight percent of patients had received ≥5 prior lines of therapy, and 36.5% had ECOG PS ≥ 2. The ORR was 35%, mPFS 2.7 months, and mOS 9.5 months, with no new safety concerns [85,86].

In the MajesTEC-1 study, the anti-BCMA-CD3 bsAb teclistamab confirmed excellent results (ORR 63%, with 39% of patients reaching a CR or better). Nonetheless, only 15% of patients included in the study were aged ≥75 years [87]. Given the high response rates, deep and potentially durable responses, good toxicity profiles, and feasible schedules, bsAbs may be valuable options in older patients. Nonetheless, efficacy and safety in older and frail patients need to be confirmed in specific studies and in real-world populations.

CAR T-cell therapy has revolutionized the treatment of relapsed and refractory hematologic malignancies. In this context, both idecabtagene vicleucel (ide–cel) and ciltacabtagene autoleucel (cilta–cel) target BCMA in MM cells [88] and were tested in RRMM patients who received at least four prior lines of therapy. The phase II KarMMa study led to the approval, by the US Food and Drug Administration (FDA) and EMA, of ide–cel [89], while the phase Ib/II CARTITUDE 1 study led to the approval of cilta–cel. A subgroup analysis of older patients treated with ide–cel in the KarMMa trial showed that 45/128 (35%) patients were aged ≥65 years and 20/128 (16%) ≥70 years, with an ORR of 84% and 90% and a PFS of 8.6 and 10.2 months, respectively. The rates of grade 3 cytokine release syndrome (CRS) and immune effector cell-associated neurotoxicity syndrome (ICANS) were also similar in both groups. These results were superimposable to those in the overall population [90].

Given the current approval limitations and the requirement for extensive prior treatment, it is less probable for older patients to be eligible for CAR T-cell therapy. In a recent retrospective electronic health record study of 4522 patients, the probability of reaching the fifth line of therapy at 8 years was much lower in older adults (20% for those aged ≥70 years vs. 35% for those aged <70 years, *P* < 0.01), while the risk of death before reaching the fifth line of therapy was higher (69% vs. 37%) [91]. Furthermore, the excellent response rate achieved with this therapy in older adults must be weighed against AEs, primarily infections, and prolonged cytopenias. A recent analysis observed that one-third of patients had an ongoing grade ≥ 3 cytopenia 4 months after infusion and that older age, higher number of prior lines of therapy, and prior history of ≥1 ASCT were significantly correlated with poor hematologic recovery 4 months after CAR T-cell therapy [92]. Nevertheless, a recent retrospective single-center experience comparing patients aged <70 years (the most represented population) vs. patients aged ≥70 years treated with anti-BCMA CAR T-cell therapy showed no differences in terms of response and toxicity, except for prolonged thrombocytopenia [93]. Ultimately, further evaluation in this patient population, including both clinical trials and real-word studies, is needed to expand the applicability of these novel therapies.

## 5. Conclusions and Future Perspectives

A substantial proportion of older patients are currently treated with new drugs and benefit from long-term disease control. However, frail patients are less able to tolerate treatment and more likely to experience AEs and disease progression. The onset and recurrence of the disease in frail patients are complicated by the presence of comorbidities and concomitant polypharmacy, which may worsen the disease and aggravate patient management. Assessing the frailty status can help guide treatment decisions and prevent higher treatment toxicity and impairments in QoL. A careful balance between efficacy and safety is essential for the optimal treatment of intermediate-fit and frail patients. Dose reduction and a dexamethasone-sparing approach can be considered if toxicity limits treatment tolerability, since there is emerging evidence of similarly effective dose-reduced regimens. The prevention and treatment of infections and the optimization of supportive care improve patients’ tolerability, reduce toxic deaths, and have consequently become mainstays in the treatment of older MM patients.

Hopefully, frailty assessment will become more manageable, widespread, and implemented in clinical practice in the future. The identification of frail patients is essential not only to guide treatment decisions, but also to tailor available treatments and design studies dedicated to frail patients. The implementation of subanalyses on quality of life and studies on patient-reported outcomes could complete the picture, helping to optimize patient management. Anti-CD38 mAbs have revolutionized the treatment of MM, and upcoming immunotherapeutic drugs, such as bsAbs, will lead to additional therapeutic advances. In the near future, we need to define and investigate the role and feasibility of new immunotherapies for older patients. In this view, clinical trials to assess the risk/benefit ratio in older and frail patients are needed.

## Figures and Tables

**Table 1:** Frailty subgroups and related outcomes in the ALCYONE and MAIA studies.

	MAIA [44,45] Dara-Rd FIT *n* = 68	ALCYONE [46] Dara-VMP FIT *n* = 48	MAIA [44,45] Dara-Rd INTER-MEDIATE *n* = 128	ALCYONE [46] Dara-VMP INTER-MEDIATE *n* = 139	MAIA [44,45] Dara-Rd FRAIL *n* = 172	ALCYONE [46] Dara-VMP FRAIL *n* = 163
**ORR**	100%	95.8%	96.9%	92.1 %	87.2%	88.3%
**mPFS**	NR	NR	NR	40.1 mo	NR	32.9 mo
**mDOT**	34.6 mo	37.2 mo	33.2 mo	36.2 mo	31.1 mo	24.7 mo
**Infections, G ≥ 3**	23.5%	12.5%	35.9%	27.5%	41.7%	30%
**Toxic deaths**	1.5%	0	4.7%	5.1%	11.9%	0
**Discontinuation due to PD**	20.6%	2.1%	19.5%	5.8%	18.6%	8.8%
**Discontinuation due to AEs**	7.4%	2.1%	7%	4.3%	9.9%	6.9%
**Discontinuation due to noncompliance with the protocol**	1.5%	0	3.9%	0.7%	4.7%	5.6%
**Median follow-up**	36.4 mo	40.1 mo	36.4 mo	40.1 mo	36.4 mo	40.1 mo

Abbreviations: Dara, daratumumab; R, lenalidomide; d, dexamethasone; V, bortezomib; M, melphalan; P, prednisone; ORR, overall response rate; mPFS, median progression-free survival; mDOT, median duration of treatment; G, grade; PD, progressive disease; AEs, adverse events; mo, months; NR, not reached.

**Table 2:** Patient characteristics in studies evaluating first-line treatment for transplant-ineligible patients with newly diagnosed multiple myeloma.

		ALCYONE [43]	MAIA [45]	SWOG S0777 [48]	Real MM [49]
**Age**	Median (years)	71	73	63	76
	≥75	30%	44%	>65 years: 43%	55%
	≥80	9%	18.5%	Not reported	>80 years: 19%
**ECOG PS**	0–1	75%	83%	86%	81%
	2	25%	17%		13%
	>2	0	0	2–3: 14%; >3: excluded	6%
**Creatinine clearance**	30–60 mL/min	41%	41%	5% creat. >2 mg/dL	40%
	<30 mL/min	Excluded (<40 mL/min)	Excluded	Excluded	9%
**Major exclusion criteria**		∘ AST/ALT > 2.5 ULN ∘ Previous malignancy < 3 years ∘ Myocardial infarction < 1 year	∘ AST/ALT > 2.5 ULN ∘ Malignancy < 5 years ∘ Myocardial infarction < 1 year	∘ Previous malignancy ∘ NYHA III–IV ∘ Recent myocardial infarction ∘ Poorly controlled diabetes ∘ Active infection	∘ Active infection ∘ Peptic ulcer

Abbreviations: R, lenalidomide; d, dexamethasone; V, bortezomib; M, melphalan; P, prednisone; ECOG PS, Eastern Cooperative Oncology Group Performance Status; creat., creatinine; AST, aspartate transaminase; ALT, alanine transaminase; ULN, upper limit of normal; NYHA, New York Heart Association classification.

**Table 3:** Ongoing trials with newer drug combinations for the treatment of transplant-ineligible patients with newly diagnosed multiple myeloma.

Clinical Trials	Regimens	Phase	Primary Endpoints
IMROZ [60]	Isa–VRd vs. VRd	III	PFS
EMN20 (NCT04096066) [61]	KRd vs. Rd	III	MRD negativity PFS
DREAMM-9 [62,63]	Belamaf VRd vs. VRd	III	MRD negativity PFS
MajesTEC-7 [64]	Tec–DR vs. DRd	III	MRD negativity PFS

Abbreviations: Isa, isatuximab; V, bortezomib; R, lenalidomide; d, dexamethasone; Belamaf, belantamab mafodotin; Tec, teclistamab; D, daratumumab; PFS, progression-free survival; MRD, minimal residual disease negativity.
